# Bellevue Hospital—Nitrate of Potash in Large Doses in Acute and Chronic Rheumatism

**Published:** 1848-12

**Authors:** 


*Bellevue Hospital.—Nitrate of Potash in Large Doses in Acute and Chronic Rheumatism.* By C. R. Gilman, M. D., Visiting Physician to the Hospital at Bellevue.

Although the fact that nitrate of potash can be taken with entire safety, in doses much larger than those to which it is restricted in the text books, was long ago established by direct and careful experiment, and although the utility of such doses in the treatment of inflammatory diseases was at a period as early confidently asserted, by those who had made trial of the remedy, yet when it was, a few years ago, re-introduced by M. Gendrin and M. Solon, with particular reference to its powers over acute rheumatism, it was received by the majority of the profession as a new remedy, and in England and this country very many were prompt in denouncing it as hazardous in the extreme. Johnson, in the *Med. Chir. Review*, says: "M. Desportes and Martin Solon are guilty of eggregrious blundering when they fancy that rheumatism may be most quickly and effectually cured by '*monstre*' doses of nitre." Denounced in such spirit the practice has never been generally adopted.

Having lately been successful with it in a few cases in private practice, I was led to make trials of it at Bellevue. Before communicating to the profession the results of these trials, it will, perhaps, be well to say a word or two on the history of these large doses of nitre. Dr. William Alexander, about 1766, undertook a variety of experiments on nitre, which he subsequently published. From the second edition of his "Experimental Essays," published in 1770, for a copy of which I am indebted to my learned colleague, Dr. John B. Beck, I find that he took (by way of experiment) so much as an ounce and a half of nitre, dissolved in a quart of water, in twenty four hours, without marked uneasiness. But Alexander found that although he was able to drink, in the course of twenty-four hours, a quart of water in which twelve drachms of nitre had been dissolved, yet when he attempted to take doses of the salt, fresh dissolved, he felt great inconvenience from drachm doses; he took eight, (each dissolved in four ounces of water,) but when he tried drachm and a half doses he was able to take but three. From these experiments he inferred that "much larger quantities of the salt may be taken than were before given, and that the power of the remedy is much greater when each dose was taken immediately after it was dissolved than when kept for hours in a state of solution." Alexander further states that after he had thus experimented on himself, he gave the remedy in several inflammatory cases with advantage, but on this part of the subject he entered into no details.

Such were the results of the carefully performed and candidly detailed experiments of Alexander. While he was thus experimenting on the effects of large doses of nitre in the healthy system, Dr. Richard Brocklesby was trying these doses in a variety of inflammatory diseases. His results were made public before those of Alexander; Brocklesby's observations having been published in 1764.

This accumulation of evidence seems to have made little impression on the medical mind; a few vague allusions to the cases of Brocklesby and the experiments of Alexander are scattered through the works on Materia Medica, but the writers still adhere to the old doses. About ten years ago M. Gendrin, of La Pitie, revived the practice of Brocklesby; the results of his trials, and of those of M. Martin Solon, were in 1841 communicated to the Academie de Medicine, Paris, and subsequently to the English public by Dr. H. Bennet, in two papers published in the London Lancet, 1844. Even at this latter date, however, the danger of fatal inflammation of the kidney or serious disease of the stomach, was strongly insisted on by some of the most enlightened of the London physicians.

With these remarks I submit the following cases:

*Case* I.—A. B., female, aged 48; had been under treatment for upwards of six months; has taken vin. sem. colchici, Tr. actea racemosa, and various other remedies, without marked effect. The nitrate was given in ℨss. doses thrice a day for two days, then ℨi was given, and the patient began to improve, the dose was continued nearly three weeks, when she was discharged cured.

Case II.—C. D., girl, aged 19; has suffered for months from sub-acute rheumatism. The remedies given to No. 1 were faithfully tried, and also *Hyd. Potass.* with *Extr. Hyosciami.* The nitrate was used in ℨi doses, and produced similar results as in No. 1.

Case. III.—E. F., man, aged 40, admitted with sub-acute rheumatism; treated with colchicum, guiac, &c. The nitrate was given in two drachm doses. He was entirely relieved when he had taken but two ounces.

It would be easy to multiply cases but they would generally be but duplications of the above.* It will be observed that most of these cases were sub-acute or chronic, this is the only respect in which they differ from the cases of Gendrin, Solon, &c. In all the cases the dose was dissolved in about half a pint of some bland fluid, it was usually given immediately after being dissolved. As to the sensible effects, there was but little variety—in a few cases, uneasiness at stomach was complained of, immediately on swallowing the medicine, and one patient had vomiting. In most of the cases free diuresis was observed, and in one or two profuse diaphoresis, while in several the remedy produced no other sensible effects than relief of the pain. The pulse was singularly affected. It often fell rapidly immediately on the dose being swallowed.

My intelligent friend, Dr. Lawrence, Assistant Physician, gave me the following note of his observations, in one case:

Pulse before the dose was swallowed, 92; in five minutes it fell to 58, and in ten to 64; after this it gradually rose to the former standard. As a permanent effect, the pulse, when the remedy was acting favorably, fell 20 or 30 beats, soon losing all the rapidity characteristic of the disease. This immediate effect of the remedy on the pulse was noted by Alexander.—*N. Y. Annalist.*

---

*Another Victim to Quackery.*—In one of the Western Medical Journals, Dr. Meeker, of Laporte, Indiana, details a case of cancer or scirrhus tumor in the breast, to which a "*cancer doctor*" first applied a blister and removed the cuticle, and then a plaster spread over with a fine white powder. The plaster occasioned great pain, but was continued on the breast eleven hours. After it had been on only two hours, the patient began to

* The remedy has been given in about twelve cases; in all but one with marked benefit.

